# Surprising intraoperative discovery of a Ladd’s band in a patient with midgut volvulus associated with intestinal malrotation: a case report

**DOI:** 10.1093/jscr/rjag150

**Published:** 2026-03-17

**Authors:** Samia Obilat, Yahya El Harras, Lina Lasri, Kaoutar Imrani, Ittimade Nassar

**Affiliations:** Department of Central Radiology, Ibn Sina Hospital, Mohammed V University, Mfadel Cherkaoui Street, Souissi District, 10100, Rabat, Morocco; Department of Central Radiology, Ibn Sina Hospital, Mohammed V University, Mfadel Cherkaoui Street, Souissi District, 10100, Rabat, Morocco; Department of Central Radiology, Ibn Sina Hospital, Mohammed V University, Mfadel Cherkaoui Street, Souissi District, 10100, Rabat, Morocco; Department of Central Radiology, Ibn Sina Hospital, Mohammed V University, Mfadel Cherkaoui Street, Souissi District, 10100, Rabat, Morocco; Department of Central Radiology, Ibn Sina Hospital, Mohammed V University, Mfadel Cherkaoui Street, Souissi District, 10100, Rabat, Morocco

**Keywords:** intestinal malrotation, midgut volvulus, Ladd’s band, computed tomography, whirlpool sign

## Abstract

Adult intestinal malrotation is a rare condition and may remain undiagnosed until the occurrence of acute complications. We report the case of a 33-year-old woman presenting with acute abdominal pain. Contrast-enhanced computed tomography (CT) demonstrated an abnormal relationship between the superior mesenteric artery and vein, right-sided small bowel loops, left-sided colonic segments and a mesenteric whirlpool sign, consistent with midgut volvulus associated with intestinal malrotation. Although CT allows reliable identification of intestinal malrotation and midgut volvulus, the precise obstructive mechanism may remain undetermined preoperatively. Surgical exploration revealed a congenital Ladd’s band crossing the duodenum and causing extrinsic duodenal obstruction. In addition, an associated midgut volvulus related to intestinal malrotation was confirmed intraoperatively. This case highlights the diagnostic value and limitations of CT in adult malrotation and emphasizes the importance of surgical exploration to identify the true obstructive mechanism.

## Introduction

Adult intestinal malrotation may remain asymptomatic or present with symptoms related to intermittent or acute duodenal obstruction and, in severe cases, midgut volvulus [1]. Its most severe complication is midgut volvulus. Computed tomography (CT) imaging is highly effective for detecting abnormal mesenteric vessel orientation, duodenojejunal malposition and the whirlpool sign, which is characteristic of volvulus [2, 3].

However, congenital remnants such as Ladd’s bands may persist into adulthood and act as obstructive structures, yet they are rarely visible on imaging. Their identification is frequently intraoperative [4–6]. This case highlights the diagnostic gap between radiological suspicion of volvulus in intestinal malrotation and the unexpected discovery of a Ladd’s band during surgery.

## Case report

A 33-year-old woman with no significant past medical history presented with acute abdominal pain associated with a biological inflammatory syndrome. Abdominal ultrasound demonstrated dilated bowel loops, raising suspicion for bowel obstruction, without identification of an underlying cause; therefore, a contrast-enhanced CT scan was subsequently performed.

### CT findings

Contrast-enhanced CT demonstrated:

- an abnormal relationship between the superior mesenteric artery and vein, with inversion of their normal orientation, consistent with intestinal malrotation;
- right-sided small bowel loops and left-sided colonic segments, consistent with intestinal malrotation;
- associated colonic dilatation, reaching 54 mm in the sigmoid colon, consistent with downstream bowel obstruction;
- two transition points consistent with a closed-loop obstruction related to volvulus;
- a mesenteric swirling pattern forming a whirlpool sign, highly suggestive of midgut volvulus.

**Figure 1:**
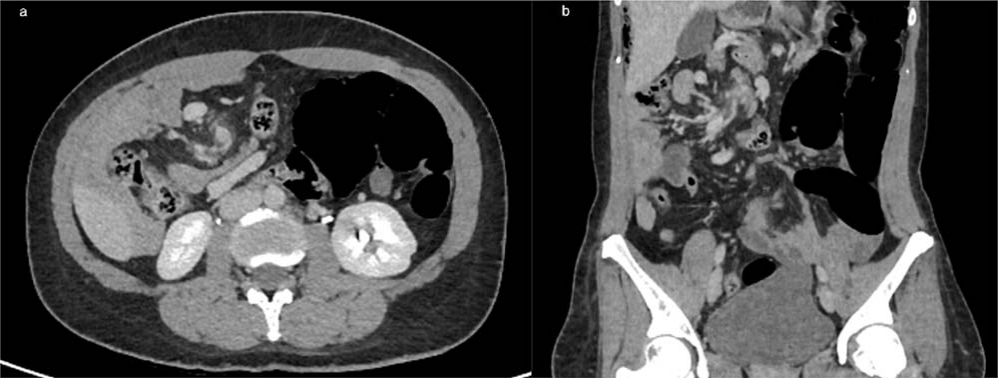
(a) Axial contrast-enhanced CT showing the classic ‘whirlpool sign,’ with clockwise swirling of the superior mesenteric vein and mesenteric fat around the SMA, consistent with midgut volvulus (b) coronal CT reconstruction confirming mesenteric torsion.

**Figure 2:**
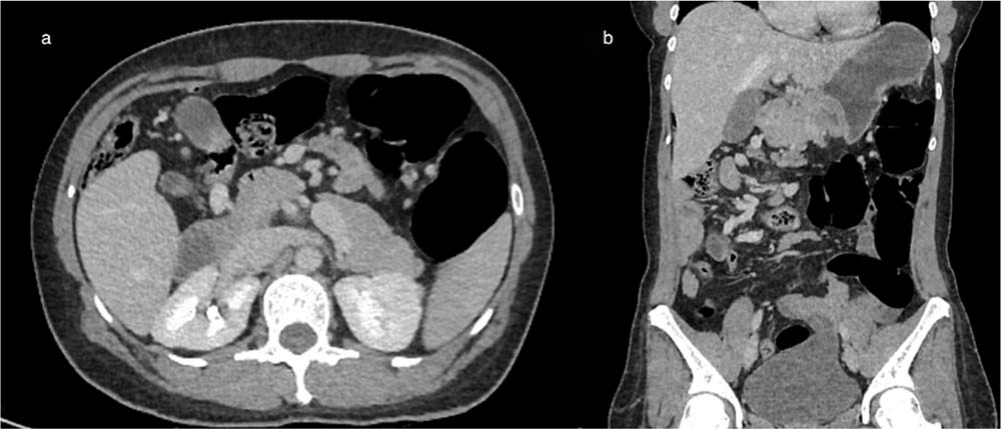
Axial (a) and coronal (b) contrast-enhanced CT images demonstrating intestinal malrotation, characterized by right-sided small bowel loops and left-sided colonic segments, with an abnormal relationship between the SMA and vein.

**Figure 3:**
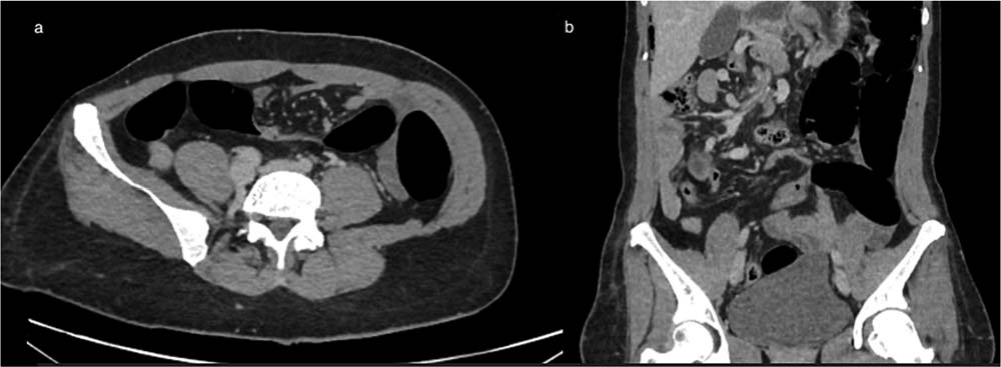
(a) Axial CT showing marked colonic distension upstream with an abrupt transition point in the right abdomen, consistent with mechanical obstruction related to volvulus (b) coronal CT reconstruction demonstrating colonic dilation and the corresponding transition zone.

These findings supported the diagnosis of midgut volvulus associated with intestinal malrotation (Figs 1–3).

Given the CT pattern of malrotation and volvulus, urgent surgical exploration was indicated.

### Intraoperative findings

Surgical exploration revealed a congenital Ladd’s band extending from the malpositioned caecum to the retroperitoneum and crossing the duodenum, responsible for extrinsic duodenal compression. In addition, intraoperative findings confirmed an associated midgut volvulus related to intestinal malrotation.

A standard Ladd procedure was performed, including division of the band, detorsion of the volvulus and broadening of the mesenteric root. All bowel segments were viable. The postoperative course was favorable, and the patient was discharged five days later.

## Discussion

Adult intestinal malrotation is rare but increasingly recognized due to the growing use of cross-sectional imaging modalities, which frequently reveal anatomical variants that previously went undiagnosed [1, 6]. Classically considered a pediatric condition, malrotation can nevertheless remain silent until adulthood. In adults, the diagnosis is often made in the setting of acute complications, the most serious of which is midgut volvulus [1].

From an embryological standpoint, malrotation results from incomplete rotation and fixation of the midgut around the superior mesenteric artery (SMA) during fetal development. Normally, the duodenojejunal junction migrates to the left upper quadrant and the caecum descends into the right lower quadrant. In malrotation, the duodenum remains on the right side of the abdomen and the caecum often lies in an ectopic position. Radiologically, this condition is characterized by an abnormal SMA–SMV relationship and the classical whirlpool sign, corresponding to twisting of the SMV and mesenteric fat around the SMA [2, 3]. In our case, CT demonstrated both the abnormal bowel distribution typical of intestinal malrotation and mesenteric swirling consistent with volvulus.

Although CT is highly sensitive for detecting volvulus and the anatomical features of intestinal malrotation, its ability to identify the underlying cause of obstruction remains limited, particularly when the mechanism is a congenital peritoneal band. Ladd’s bands are thin fibrous structures extending from the malpositioned caecum to the retroperitoneum and crossing the duodenum, causing extrinsic duodenal compression. In adults, they are rarely visualized on CT because of their thin morphology and the absence of associated inflammatory changes, and are therefore usually identified intraoperatively [4, 5]. Ladd’s bands classically cause extrinsic duodenal obstruction and may be associated with midgut volvulus [4].

The clinical implications of Ladd’s bands in adults are significant. Although classically encountered in infants, they may persist silently for decades before becoming symptomatic. Their rarity in adulthood contributes to diagnostic uncertainty and may lead clinicians to favor more common causes of bowel obstruction such as adhesions or hernias. In our patient, a Ladd’s band caused extrinsic duodenal obstruction, and midgut volvulus was found intraoperatively as an associated complication of intestinal malrotation.

Multiplanar reconstruction imaging further enhances diagnostic confidence by improving visualization of the spatial relationship between the SMA, SMV and mesenteric fat. In our case, axial and coronal reconstructions were crucial to confirming mesenteric torsion. Nevertheless, even with modern CT technology, congenital bands remain difficult to detect preoperatively, reinforcing the need for surgical exploration in severe or ambiguous clinical situations.

Another important consideration is the management of malrotation in adults. Although some patients may remain asymptomatic for years, the potential for life-threatening complications such as volvulus, bowel ischaemia and necrosis supports surgical treatment once the diagnosis is established [6]. The Ladd procedure remains the standard approach, combining division of Ladd’s bands, detorsion of volvulus when present and widening of the mesenteric root to reduce the risk of recurrence. The favorable outcome in our patient further supports the effectiveness of this strategy.

Finally, this case adds to the literature by highlighting that even when CT findings strongly suggest intestinal malrotation complicated by midgut volvulus, the precise obstructive mechanism may only be identified during surgery. Increased awareness of Ladd’s bands in adult patients is essential, particularly when no alternative cause of obstruction is evident on imaging.

## Conclusion

This case underlines the possible association between midgut volvulus and congenital fibrous bands in adults. While imaging accurately diagnosed midgut volvulus associated with intestinal malrotation, intraoperative exploration revealed a Ladd’s band responsible for extrinsic duodenal obstruction, with midgut volvulus identified as an associated complication. Radiologists should consider this possibility when encountering volvulus associated with malrotation, even when no clear obstructive structure is visible on CT. This case also highlights the importance of interdisciplinary collaboration, as the integration of radiological findings with intraoperative assessment allows accurate identification of the true obstructive mechanism and guides appropriate management.

## References

[1] Emanuwa OF, Ayantunde AA, Davies TW. Midgut malrotation first presenting as acute bowel obstruction in adulthood: a case report and literature review. World J Emerg Surg 2011;6:22. 10.1186/1749-7922-6-22 21801417 PMC3158108

[2] Nichols DM, Li DK. Superior mesenteric vein rotation: a CT sign of midgut malrotation. AJR Am J Roentgenol 1988;141:707–10. 10.2214/ajr.141.4.707 6604422

[3] Stewart ET, Pickhardt PJ, Hanson JF. Malrotation in adults: imaging findings in 12 patients. AJR Am J Roentgenol 1976;126:358–63. 10.2214/ajr.126.2.358 175705

[4] Grassi C, Conti L, Palmieri G et al. Ladd’s band in the adult: an unusual case of occlusion. Int J Surg Case Rep 2020;71:45–9. 10.1016/j.ijscr.2020.04.046 32438336 PMC7240054

[5] Moldrem AW, Papaconstantinou H, Broker H et al. Late presentation of intestinal malrotation: an argument for elective repair. World J Surg 2008;32:1426–31. 10.1007/s00268-008-9490-3 18347850

[6] Nezami N, Fang J, Oskouian RJ. Midgut volvulus in adults: CT features and the importance of the whirlpool sign. Radiol Case Rep 2019;14:1298–302. 10.1007/s10140-009-0816-8

